# The Incidence and Risk Factors of Urinary Tract Infection in Patients with Type 2 Diabetes Mellitus Using SGLT2 Inhibitors: A Real-World Observational Study

**DOI:** 10.3390/medicines9120059

**Published:** 2022-11-22

**Authors:** Suriyon Uitrakul, Krittika Aksonnam, Pimchanok Srivichai, Sorawit Wicheannarat, Supatcha Incomenoy

**Affiliations:** Department of Pharmaceutical Care, Walailak University, Tha Sala, Nakhon Si Thammarat 80160, Thailand

**Keywords:** incidence, risk factor, type 2 diabetes mellitus, sodium glucose co-transporter-2 inhibitor, urinary tract infection

## Abstract

**Background**: The incidence and risk of urinary tract infection (UTI) in patients with type 2 diabetes mellitus (T2DM) who use sodium glucose co-transporter-2 (SGLT2) inhibitors are still controversial. Therefore, this study aimed to investigate the incidence and risk factors of using SGLT2 inhibitors, particularly in Thai patients. **Methods**: Electronic medication records of all patients, who started the treatment of T2DM between 1 January 2019 and 30 June 2021 at a tertiary hospital in Thailand, were reviewed. The patients were divided into SGLT2 inhibitor and non-SGLT2 inhibitor groups to compare the incidence of UTI. **Results:** The overall incidence rate of UTI was 33.49% in the SGLT2 inhibitor group and 11.72% in the non-SGLT2 inhibitor group. The incidence rates of UTI were not different between dapagliflozin and empagliflozin treatment (34.00% and 33.03%, respectively). Patients treated with SGLT2 inhibitors had a 3.70 higher risk of UTI compared with those treated with non-SGLT2 inhibitors (95%CI 2.60–5.29). Moreover, the significant risk factors for UTI found in this study were gender, age, and occupation. **Conclusions:** This study highlighted the high incidence of UTI in patients using dapagliflozin and empagliflozin compared with non-SGLT2 inhibitors. Additionally, patients of female gender and older age had a significantly higher risk of UTI when treated with SGLT2 inhibitors, whereas those with permanent jobs had a lower risk.

## 1. Introduction

Sodium glucose co-transporter 2 (SGLT2) inhibitors are a novel class of oral anti-hyperglycemic agents that are used in the treatment of type 2 diabetes mellitus. The mechanism of SGLT2 inhibitors involves the inhibition of glucose reabsorption by the kidney, increasing glucose excretion and lowering blood glucose levels in patients with diabetes [1]. However, according to some studies, there were several reported adverse events associated with the use of SGLT2 inhibitors, such as urinary tract infection (UTI) and polyuria.

In 2015, the Food and Drug Administration of the United States (US FDA) launched a warning about the risks of serious UTI due to SGLT2 inhibitors [2]. In patients with type 2 diabetes, UTI is a known prevalent complication. A study by Hirji et al. described that the incidence of UTI among patients with diabetes was 46.9 per 1000 person-years (95%CI 45.8–48.1), whereas among people without diabetes it was 29.9 per 1000 person-years (95%CI 28.9–30.8) [3]. However, several studies reported a higher incidence of UTI in such patients using SGLT2 inhibitors.

Several meta-analysis studies reported that patients with type 2 diabetes mellitus who used SGLT2 inhibitors had a significantly higher risk of UTI than those who used a placebo or other oral anti-diabetic agents [4,5]. Conversely, some meta-analysis studies concluded that there was no significant difference in the risk of UTI between patients with SGLT2 inhibitors and placebo [6,7]. As a result, the risk of UTI caused by SGLT2 inhibitors is still controversial.

Furthermore, there was no report of this drug-induced UTI in Thailand. In Thailand, UTI is known to be one of the diseases that many patients tend not to go to hospitals with, because the antibiotics for the treatment of UTI can be prescribed by pharmacists in drug stores. Therefore, the underreported cases of UTI are feasible if the events are observed using only urinary analysis results. This study aimed to investigate the overall incidence of UTI related to SGLT2 inhibitors in Thai patients with type 2 diabetes mellitus, as well as its potential risk factors.

## 2. Materials and Methods

### 2.1. Patient Population

This study was a retrospective cohort study. The population was divided into two groups: the study group (patients using SGLT2 inhibitors) and the control group (patients using non-SGLT2 inhibitors). The inclusion criteria were patients with the diagnosis of type 2 diabetes mellitus who were treated at Maharaj Nakhon Si Thammarat Hospital. The patients had to be first prescribed at least one oral anti-diabetic agent, either SGLT2 inhibitors or non-SGLT2 inhibitors, during the period of 1 January 2019 to 30 June 2021. The exclusion criteria were patients who used insulin, had incomplete data, passed away during the study, or refused to participate in the study by telephone. The incomplete data were defined as any participants with only the first visit for treatment (pre-treatment) or without the first visit for treatment. In addition, patients with data indicating urinary tract infection at pre-treatment were excluded.

### 2.2. Study Design

The data collection in this study was conducted in two parts. The first part was an electronic medication record (EMR) review. The EMRs of all recruited patients were reviewed in order to collect all information, including gender, age, religion, occupation, weight, height, body temperature, laboratory results, and prescribed anti-diabetic agents, as well as concurrent medication. The relevant laboratory data included parameters in the blood (i.e., Hemoglobin A1c (Hb_A1c_), fasting blood sugar (FBS), and serum creatinine (SCr), and parameters in urine including bacterial count, white blood cell (WBC) count, and urinary glucose. Additionally, the diagnosis of UTI in the hospital with the following ICD-10 codes was recorded: N39 indicates urinary tract infection with the site not specified; N30 indicates acute cystitis; N34 indicates urethritis and urethral syndrome; N10 indicates acute tubulointerstitial nephritis.

The second part was data collection via direct telephone calls to the recruited patients in order to inquire about their histories of UTI. This ensured that as many as UTI events as possible were collected even though the patients had never been to the hospital due to UTI symptoms. The questions consisted of two sections. The first section asked whether the patient was ever diagnosed with UTI outside the hospital (e.g., at a clinic or a pharmacy store) after treatment with oral anti-diabetic agents. The second part asked whether the patient used to have any symptoms indicating UTI. These included acute dysuria, fever, urinary incontinence, frequency, suprapubic pain, hematuria, or pain in the lumbar region (costovertebral angle tenderness). If the patients answered positively for either of the two sections, they were asked to describe the time of the event if possible.

### 2.3. Outcome Measurement

The primary outcome of this study was the first time that patients had UTI after treatment with anti-diabetic agents. An event of UTI in this study was identified if a patient had one of the following criteria. Firstly, a patient was diagnosed by a doctor at Maharaj Nakhon Si Thammarat hospital with the ICD-10 codes N39, N30, N34, and N10. Secondly, a patient had at least one of these laboratory results post-treatment: (1) reports of bacteria in urine were few, moderate, or severe; or (2) urinary leukocytes > 10 cell/mm^3^. Thirdly, a patient had acute dysuria with or without fever, with at least one of these symptoms: urinary incontinence, frequent urination, suprapubic pain, hematuria, and pain in the lumbar region. Lastly, a patient had the diagnosis of UTI at a medical clinic or a pharmacy store.

The secondary outcome of this study was the time between the first drug use and the first UTI occurrence, and the risk factors of UTI investigated from patient characteristics and relevant laboratory data, including age, gender, body mass index (BMI), religion, occupation, and the laboratory results regarding levels of substances in the blood (i.e., Hb_A1c_, FBS, and SCr).

### 2.4. Statistical Analysis

Baseline characteristics of all patients were descriptively analyzed and reported as numbers, means, and percentages. The difference in the incidence of UTI between patients treated with SGLT2 inhibitors and non-SGLT2 inhibitors was analyzed using the Chi-Square test. The time to event was analyzed using the Kaplan-Meier curve with the Log-rank test. The odds ratio (OR) of the UTI events and the expected relevant risk factors were investigated using Logistic regression analysis. All the statistical analyses were performed using IBM SPSS Statistics software version 28.0.0.0. (IBM, New York, NY, USA). The significance threshold of all analyses was set at *p* < 0.05.

## 3. Results

### 3.1. Patient Characteristics

Between 1 January 2019 and 30 June 2021, there were 853 patients with diabetes mellitus who met the inclusion criteria and were recruited for analysis. Table 1 describes the baseline characteristics of all included patients. Of these, 418 patients (49.00%) were in the SGLT2 inhibitor group, divided into 200 (47.85%) patients using dapagliflozin and 218 (52.15%) patients using empagliflozin. There were 435 patients (51.00%) in the non-SGLT2 inhibitor group. In the SGLT2 inhibitors group, 234 cases (55.98%) were female; the average age and BMI were 63.67 years and 26.51 kg/m^2^, respectively. In the other group, 144 cases (33.10%) were female, with an average age of 55.68 years and an average BMI of 25.78 kg/m^2^. Buddhism was the most common religion, and Metformin and Sulfonylurea were the two most common oral anti-diabetic drugs in both groups.

Laboratory results of both groups are also shown in Table 1. The mean baseline Hb_A1c_ in the SGLT2 inhibitors group was not different from the non-SGLT2 inhibitors group (8.6 and 8.73%, respectively). The mean baseline FBS was also similar between both groups (164.32 and 174.18 mg/dL, respectively). Moreover, the mean baseline serum creatinine in patients treated with SGLT2 inhibitors was 0.97 mg/dL, similar to 0.86 mg/dL in patients with non-SGLT2 inhibitors. Statistical analysis of the baseline characteristics is shown in Supplementary Table S1.

### 3.2. The Incidence of UTI

**Figure 1:**
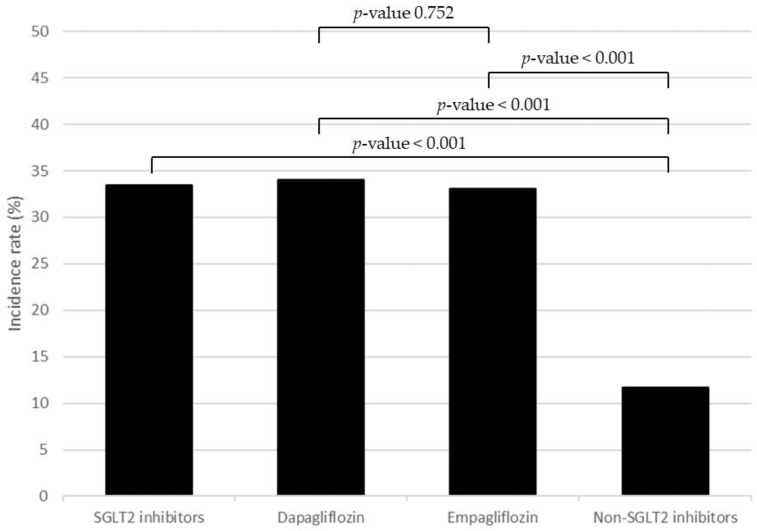
The incidence rate of UTI compared between SGLT2 inhibitors, dapagliflozin, empagliflozin, and non-SGLT2 inhibitors.

The overall incidence rate of UTI in the SGLT2 inhibitor group was 33.49%, divided into 34.00% for dapagliflozin and 33.03% for empagliflozin, respectively, whereas the incidence rate of UTI in the non-SGLT2 inhibitor group was 11.72% (Figure 1). The incidence rate of patients who used SGLT2 inhibitors was significantly higher than those who used non-SGLT2 inhibitors (*p*-value < 0.001). In addition, the UTI incidence rate of patients using dapagliflozin was not significantly different from that for patients using empagliflozin. Moreover, compared with patients using non-SGLT2 inhibitors, patients who were treated with SGLT2 inhibitors had a 3.71 times higher UTI risk (95%CI 2.60–5.29) (Table 2).

**Figure 2:**
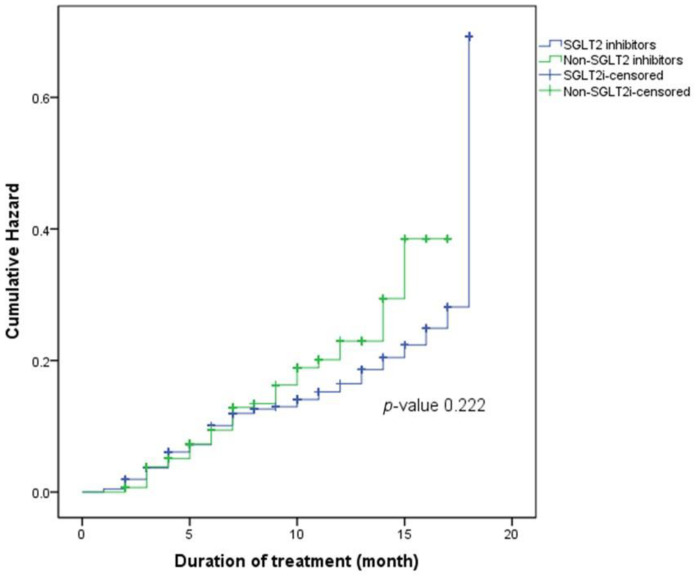
Kaplan-Meier curve of the cumulative hazards of UTI in patients who used SGLT2 inhibitors and non-SGLT2 inhibitors.

Regarding the time of UTI events, the mean time to UTI event of patients in the SGLT2 inhibitor group was 15.92 months, and that of patients in the non-SGLT2 inhibitor group was 14.69 months. The Kaplan-Meier curve in Figure 2 shows the cumulative incidence of the patients in both groups, and the Log-rank test indicated no significant difference among them (*p*-value 0.222).

### 3.3. The Factors Associated with UTI

The results indicated that gender and occupation were the risk factors associated with UTI (Table 3). In particular, the female gender had a 1.75 times higher risk than the male gender (*p*-value 0.031). On the other hand, permanent employees or salarymen had a 0.55 times (95%CI 0.30–0.99) decrease in UTI risk compared with people with other jobs. Other factors were not associated with UTI events, including age, BMI, religion, blood sugar levels, and serum creatinine.

The odd ratio of using SGLT2 inhibitors that was adjusted with the relevant risk factors was 4.85 (95% CI 2.77–8.52). The treatments with dapagliflozin and empagliflozin delivered risks that were 4.23 and 5.29 times higher than treatment with non-SGLT2 inhibitors, respectively (Table 2).

## 4. Discussion

The results of this study indicated an increase in UTI occurrence in patients who used SGLT2 inhibitors, i.e., dapagliflozin and empagliflozin. The UTI incidence in patients who used SGLT2 inhibitors was more than 30% compared with 12% in those who used non-SGLT2 inhibitors. In other words, using SGLT2 inhibitors increased the risk of UTI 3.7 times more than using non-SGLT2 inhibitors. Moreover, the factors that were associated with UTI in this study were gender and occupation.

The incidences of UTI that were reported in this study were much higher than in several previous studies; the UTI incidences related to SGLT2 inhibitors were usually approximately reported at 3–9% [8,9]. Focusing on each drug, most other studies also reported lower incidences than the current study. For instance, in patients using dapagliflozin, the reported incidence of UTI was 5.3% [10], compared with 34% in this study. Likewise, 4% of patients who used empagliflozin had reported UTI events in other studies [10,11], but 33% of such patients had the same incidence in this study.

The same phenomenon was observed when considering odds ratios of UTI. Patients who used SGLT2 inhibitors in this study tended to have a more than 3.70 times increase in the risk of UTI, compared with patients with non-SGLT2 inhibitors. In contrast, many meta-analysis studies reported the increased risk of UTI ranged between 1.10 to 1.30 times, compared with placebo [4,5]. Moreover, several studies concluded that SGLT2 inhibitors did not significantly increase the risk of UTI when compared with placebo or other anti-diabetes agents [6,7].

Focusing on an individual SGLT2 inhibitor, dapagliflozin has shown increases in UTI risk in many studies. For instance, the study by Figueiredo et al. reported a significant increase in UTI of 1.18-fold, similar to the study by Puckrin et al. that reported a 1.33-fold increase [4,6]. Although this study found that patients treated with dapagliflozin had a significant increase in UTI risk, the results were much higher than in most published studies. Moreover, this study found a significant increase in UTI events in patients treated with empagliflozin, whereas other meta-analysis studies concluded that there was no significance [12].

The main cause of differences in the incidence and odds ratio between this study and the previous studies might be the difference in data collection; the above-mentioned studies included only UTIs documented in hospitals, whereas in the current study, all types of UTIs were recorded. In addition, the difference in diagnostic criteria, such as signs, symptoms, self-report, and urine culture, could result in a dissimilar incidence of UTIs [11,13]. The majority of the studies used only urinary laboratory results to diagnose the events of UTI, which were different from the current study, which diagnosed UTI based on wider criteria, including inside (i.e., urinary analysis results) and outside the hospital setting (i.e., patient symptoms and diagnosis in medical clinics or pharmacy stores). These methods of event collection, therefore, resulted in higher reported incidences of UTI than other studies.

The factors affecting UTI events have been widely studied, and the results in this study showed the same results. Female gender is one of the known risk factors of UTI; many studies indicated that females had higher events suggestive of UTI and had a greater tendency for developing symptomatic UTI and recurrent complications with a higher incidence of more serious complications than the male population [14,15].

Another factor that was found to be associated with UTI in the present study was being a permanent employee. Even though there are not many studies that indicated an association between occupation and UTI, some jobs were considered to be the risk factors of UTI, such as cleaning and driving taxis or buses [16,17]. On the other hand, this study observed a positive impact of having permanent employment, such as among salarymen or civil servants, on the reduction in UTI risk. A hypothesis for this might be the higher education of general salarymen and civil servants than farmers and laborers; consequently they should have better personal hygiene, including the hygiene of the urinary system. Nevertheless, this hypothesis needs to be further studied.

According to the results of this study, other factors were not associated with the incidence of UTI, including age, BMI, religion, FBS, Hb_A1c_, and serum creatinine. However, unlike the present study, there were reports of increasing UTI events of some factors. For example, an elevated BMI, especially more than 30 kg/m^2^, was associated with an increased incidence of UTI because of the inability to exert sufficient pressure to empty the urinary bladder [18]. Elevated glycosylated hemoglobin was also a predisposal factor for UTI in patients with diabetes mellitus [19].

As this study was conducted retrospectively, there were several limitations that should be highlighted. First, the collection of data from both laboratory records in the hospital to identify the UTI events might be underreported because some patients did not go to hospitals. Additionally, patients with long-term diabetes mellitus have a high risk of neurogenic bladder, so they might not recognize their symptoms of UTI and might not go to the hospital. In fact, all patients with uncontrolled diabetes mellitus should be monitored for neurogenic bladder, and if they have the disease, there are several treatments that can be used [20]. Moreover, the questionnaire via phone calls might be affected by recall bias. As the patients were asked to answer questions about the events of UTI after diabetic treatment, some patients might be confused that the symptoms that occurred before or after the drug use. In addition, this was the reason that this study could not recruit patients who started the anti-diabetic drugs more than two years previously because their answers might not be reliable.

The second limitation was the criteria for the diagnosis of UTI in this study, which were different from those used in the previous studies [11,13], and therefore could result in a higher reported incidence of UTI. However, the criteria that were used in this study were considered the minimum criteria for the diagnosis of UTI and were widely used in pharmacy stores or medical clinics where urinary analysis is not possible [21,22]. Thirdly, this study did not adjust for other factors, such as urinary glucose, concurrent medication, or the personal hygiene of the patients, because of the incomplete data in patient medical records. Therefore, there might still be several confounding factors that interfered with the reported incidence of UTI, such as poor hygiene and functional abnormalities of the urinary tract of some patients. The implication of the current findings should be interpreted in conjunction with clinical expert judgements.

## 5. Conclusions

The incidence of UTI in patients with SGLT2 inhibitor treatment was 33.49%, compared with 11.72% in patients without SGLT2 inhibitor treatment. In addition, the results indicated that dapagliflozin and empagliflozin were associated with significant increases in UTI risk, by 3.79 and 3.64 times, respectively, compared with non-SGLT2 inhibitors. Female patients should be closely monitored for UTI events, and patients who work as permanent employees might be safer when treated with SGLT2 inhibitors.

## Figures and Tables

**Table 1:** Demographic and clinical characteristics of patients who received SGLT2 inhibitor and non-SGLT2 inhibitor therapy.

Characteristic	SGLT2 Inhibitors			Non-SGLT2 Inhibitors
	Dapagliflozin	Empagliflozin	Total	
	(*n* = 200)	(*n* = 218)	(*n* = 418)	(*n* = 435)
Gender, *n* (%)				
Male	70 (35.00)	114 (52.29)	184 (44.02))	291 (66.90)
Female	130 (65.00)	104 (47.71)	234 (55.98)	144 (33.10)
Age, mean (95%CI) (year) <40 years, *n* (%) 40–49 years, *n* (%) 50–59 years, *n* (%) ≥60 years, *n* (%)	61.96 (60.99–64.35) 4 (2.00) 18 (9.00) 51 (25.50) 127 (63.50)	65.23 (64.13–67.33) 1 (0.46) 12 (5.50) 49 (22.48) 156 (71.56)	63.67 (63.15–69.50) 5 (1.20) 30 (7.17) 100 (23.92) 283 (67.7)	55.68 (53.84–57.20) 43 (9.89) 90 (20.69) 133 (30.57) 169 (38.85)
BMI, mean (95%CI) (kg/m^2^) <30 mg/m^2^, *n* (%) ≥30 mg/m^2^, *n* (%)	26.95 (25.60–27.07) 156 (78.00) 44 (22.00)	26.11 (25.57–26.81) 182 (83.49) 36 (16.51)	26.51 (25.78–26.73) 338 (80.86) 80 (19.14)	25.78 (25.02–26.43) 361 (82.99) 74 (17.01)
Religion, *n* (%)				
Buddhism	193 (96.50)	203 (93.12)	396 (94.74)	397 (91.26)
Islam	2 (1.00)	1 (0.46)	3 (0.72)	18 (4.14)
Others	5 (2.50)	14 (6.42)	19 (4.55)	20 (4.60)
Occupation, *n* (%)				
Permanent employee	86 (43.00)	87 (39.91)	173 (41.39)	74 (17.01)
Temporary workers	38 (19.00)	42 (19.27)	80 (19.14)	195 (44.83)
Agricultural jobs	28 (14.00)	24 (11.01)	52 (12.44)	72 (16.55)
Others	48 (24.00)	65 (29.82)	113 (27.03)	94 (21.61)
Hb_A1c_, mean (95%CI) (%)	8.60 (8.26–8.87)	8.58 (8.36–8.80)	8.60 (8.39–8.79)	8.73 (8.43–9.00)
FBS, mean (95%CI) (mg/dL)	162.31 (154.49–170.13)	166.24 (158.84–173.64)	164.32 (158.97–169.68)	174.18 (167.69–180.67)
Serum creatinine, mean (95%CI) (mg/dL)	0.92 (0.85–0.96)	1.02 (0.96–1.06)	0.97 (0.92–0.99)	0.86 (0.82–0.88)
Type of oral anti-diabetic drugs *, *n* (%)				
Metformin	187 (93.50)	186 (85.32)	373 (89.23)	435 (100.00)
Sulfonylurea	136 (68.00)	124 (56.88)	260 (62.20)	161 (37.01)
Pioglitazone	71 (35.50)	63 (28.90)	134 (32.06)	43 (9.89)
DPP-4 inhibitors	42 (21.00)	46 (21.10)	88 (21.05)	2 (0.46)
Others	14 (7.00)	20 (9.17)	34 (8.13)	20 (4.60)

* Each patient could have more than one. Abbreviation: SGLT2, sodium glucose co-transporter type 2; BMI, body mass index; Hb_A1c_, hemoglobin A1c; FBS, fasting blood sugar; DPP-4, dipeptidyl peptidase-4.

**Table 2:** Odds ratios of overall urinary tract infection in patients treated with SGLT2 inhibitors compared with patients treated with non-SGLT2 inhibitors.

Drug	Odds Ratio (95%CI)	Adjusted Odds Ratio (95%CI)
Non-SGLT2 inhibitors	Reference	Reference
SGLT2 inhibitors	3.71 (2.60–5.29)	4.85 (2.77–8.52)
Dapagliflozin	3.79 (2.51–5.71)	4.23 (2.18–8.23)
Empagliflozin	3.64 (2.42–5.44)	5.29 (2.76–10.27)

Note: Odds ratios were adjusted for age, gender, BMI, religion, occupation, Hb_A1c_, FBS, and SCr. Abbreviation: SGLT2, sodium glucose co-transporter type 2.

**Table 3:** The factors associated with UTI events in patients who were treated with SGLT2 inhibitors.

Variable	Odds Ratio (95%CI)	*p*-Value
Gender		
Male	0.57 (0.34–0.95)	0.031
Female	Reference	
Age		
<40 years	1.64 (0.55–4.89)	0.379
40–49 years	1.68 (0.78–3.60)	0.182
50–59 years	1.30 (0.73–2.30)	0.376
≥60 years	Reference	
Body mass index		
<30 mg/m^2^	0.60 (0.34–1.05)	0.073
≥30 mg/m^2^	Reference	
Religion		
Buddhism	1.46 (0.46–4.64)	0.522
Islam	2.15 (0.28–16.59)	0.463
Others	Reference	
Occupation		
Permanent employee	0.55 (0.30–0.99)	0.046
Temporary workers	0.73 (0.39–1.35)	0.308
Agricultural jobs	0.84 (0.40–1.76)	0.647
Others	Reference	
Hemoglobin A1c	0.97 (0.83–1.12)	0.652
Fasting blood sugar	0.99 (0.99–1.00)	0.821
Serum creatinine	0.87 (0.37–2.07)	0.755

## Data Availability

The data are available from the corresponding authors upon request.

## Supplementary Materials

The following supporting information can be downloaded at: https://www.mdpi.com/article/10.3390/medicines9120059/s1, Table S1: Statistical analysis of baseline characteristics of patients in the SGLT2 inhibitors group and non-SGLT2 inhibitors groups.
