# Supplementary material for: The Incidence and Risk Factors of Urinary Tract Infection in Patients with Type 2 Diabetes Mellitus Using SGLT2 Inhibitors: A Real-World Observational Study
Source: Medicines (Basel). 2022 Nov 22;9(12):59. doi: 10.3390/medicines9120059 (PMC9785475; doi:10.3390/medicines9120059)
Supplement: Supplementary file 1 [file medicines-09-00059-s001.zip › medicines-2042059-supplementary.pdf]

Supplementary Table S1. Statistical analysis of baseline characteristics of patients in the SGLT2 inhibitors group and non-SGLT2 inhibitors groups

Characteristic	SGLT2 inhibitors			Non-SGLT2 inhibitors	P-value
	Dapagliflozin	Empagliflozin	Total		
	(n=200)	(n=218)	(n=418)	(n=435)	
Gender, n (%)					<0.001
Male	70 (35.00)	114 (52.29)	184 (44.02)	291 (66.90)	
Female	130 (65.00)	104 (47.71)	234 (55.98)	144 (33.10)	
Age, mean (95%CI) (year)	61.96 (60.99-64.35)	65.23 (64.13-67.33)	63.67 (63.15-69.50)	55.68 (53.84-57.20)	<0.001
< 40 years, n (%)	4 (2.00)	1 (0.46)	5 (1.20)	43 (9.89)	
40-49 years, n (%)	18 (9.00)	12 (5.50)	30 (7.17)	90 (20.69)	
50-59 years, n (%)	51 (25.50)	49 (22.48)	100 (23.92)	133 (30.57)	
≥ 60 years, n (%)	127 (63.50)	156 (71.56)	283 (67.7)	169 (38.85)	
BMI, mean (95%CI) (kg/m^2)	26.95 (25.60-27.07)	26.11 (25.57-26.81)	26.51 (25.78-26.73)	25.78 (25.02-26.43)	0.370
<30 mg/m^2, n (%)	156 (78.00)	182 (83.49)	338 (80.86)	361 (82.99)	
≥ 30 mg/m^2, n (%)	44 (22.00)	36 (16.51)	80 (19.14)	74 (17.01)	
Religion, n (%)					0.505
Buddhism	193 (96.50)	203 (93.12)	396 (94.74)	397 (91.26)	

Islam	2 (1.00)	1 (0.46)	3 (0.72)	18 (4.14)	
Others	5 (2.50)	14 (6.42)	19 (4.55)	20 (4.60)	
Occupation, n (%)					<0.001
Permanent employee	86 (43.00)	87 (39.91)	173 (41.39)	74 (17.01)	
Temporary workers	38 (19.00)	42 (19.27)	80 (19.14)	195 (44.83)	
Agricultural jobs	28 (14.00)	24 (11.01)	52 (12.44)	72 (16.55)	
Others	48 (24.00)	65 (29.82)	113 (27.03)	94 (21.61)	
Hb_{A1c}, mean (95%CI) (%)	8.60 (8.26-8.87)	8.58 (8.36-8.80)	8.60 (8.39-8.79)	8.73 (8.43-9.00)	0.808
FBS, mean (95%CI) (mg/dL)	162.31 (154.49-170.13)	166.24 (158.84-173.64)	164.32 (158.97-169.68)	174.18 (167.69-180.67)	0.188
Serum creatinine, mean (95%CI) (mg/dL)	0.92 (0.85-0.96)	1.02 (0.96-1.06)	0.97 (0.92-0.99)	0.86 (0.82-0.88)	0.005
Type of oral anti-diabetic drugs*, n (%)					0.538
Metformin	187 (93.50)	186 (85.32)	373 (89.23)	435 (100.00)	
Sulfonylurea	136 (68.00)	124 (56.88)	260 (62.20)	161 (37.01)	
Pioglitazone	71 (35.50)	63 (28.90)	134 (32.06)	43 (9.89)	
DPP-4 inhibitors	42 (21.00)	46 (21.10)	88 (21.05)	2 (0.46)	
Others	14 (7.00)	20 (9.17)	34 (8.13)	20 (4.60)	

Note: The comparison was between patients who used SGLT2 inhibitors and non-SGLT2 inhibitors. Categorical variables were compared using Chi-square test, and continuous variables were compared using Mann-Whitney U test.
